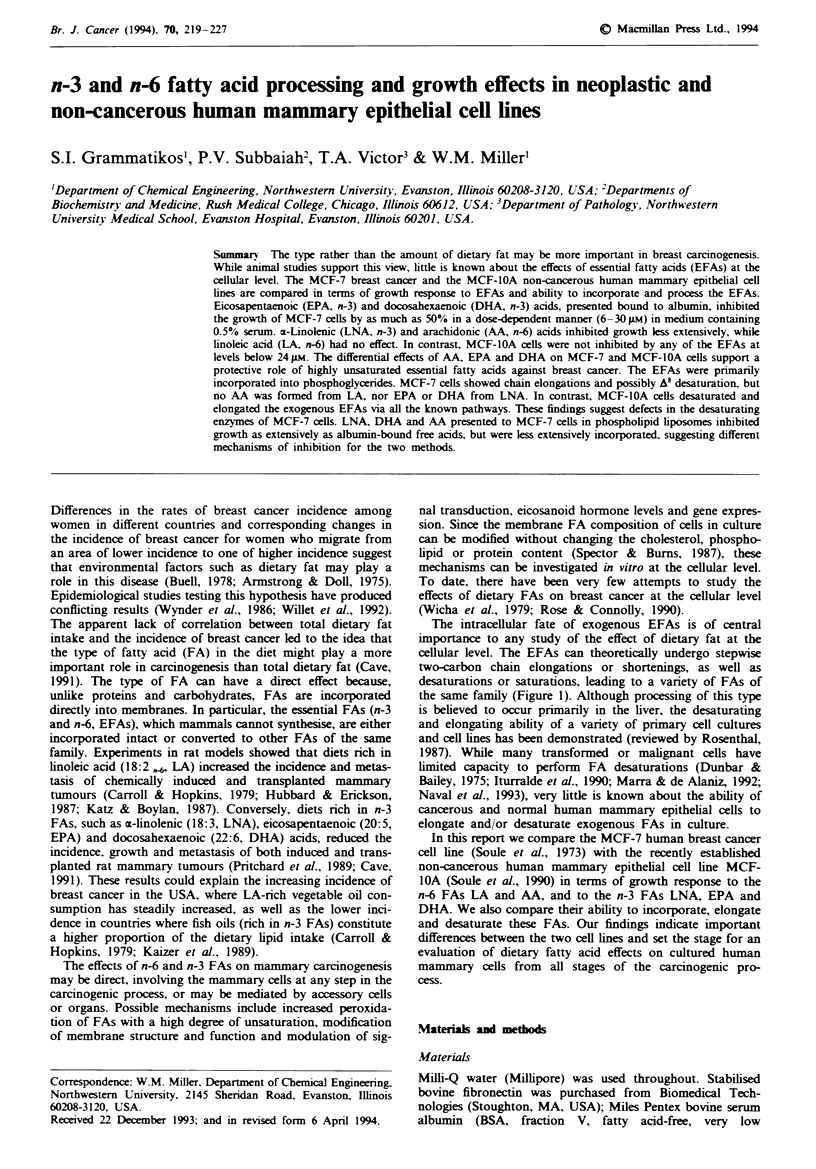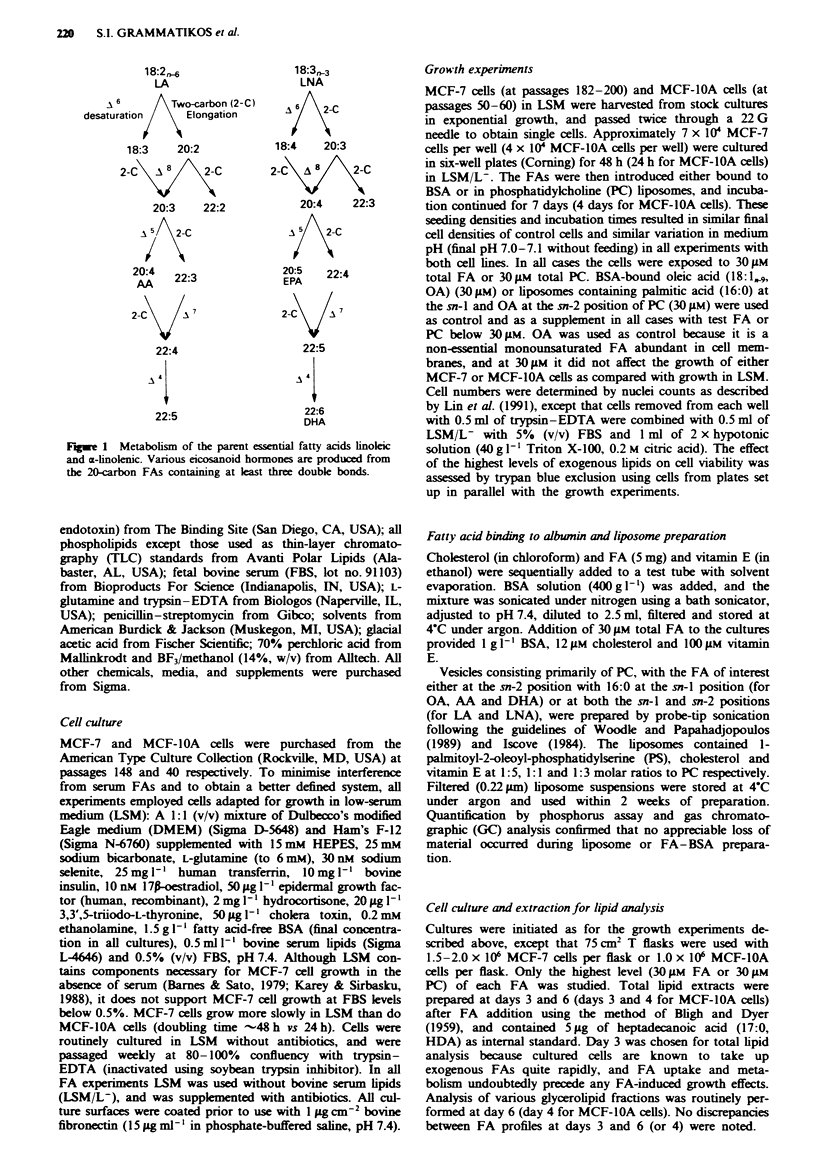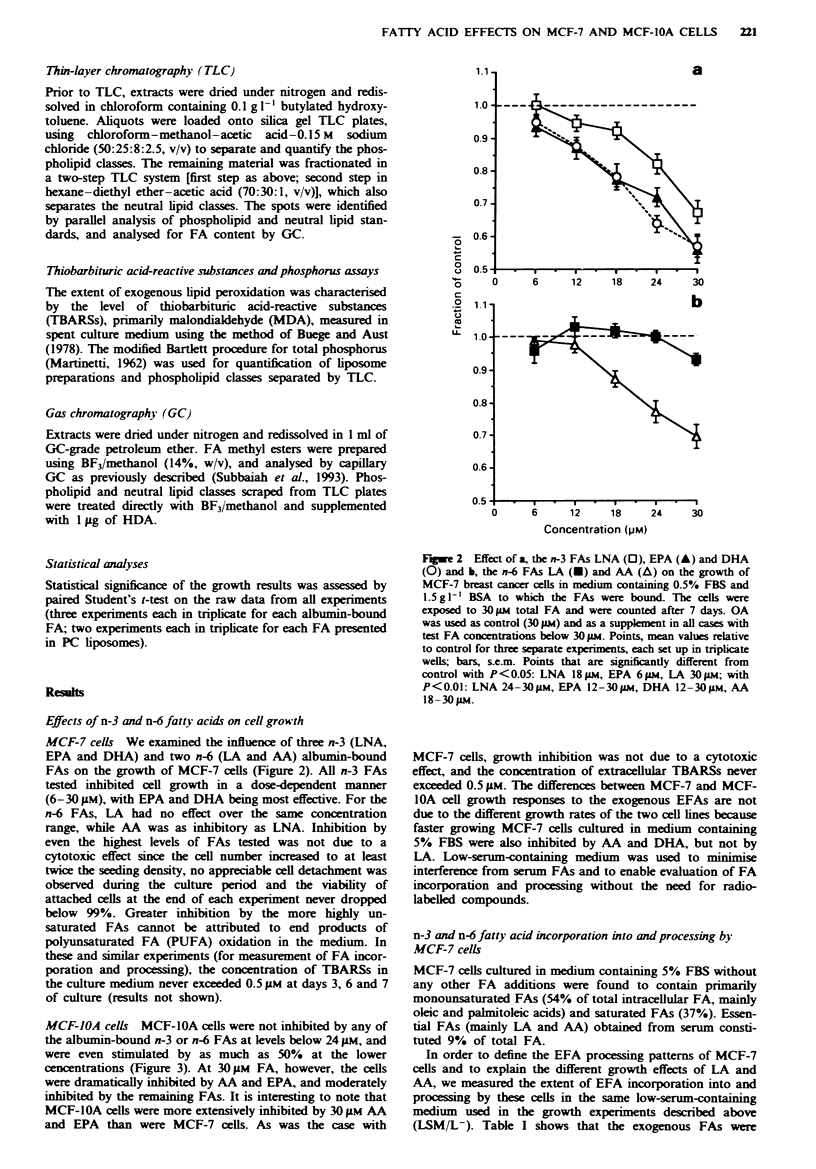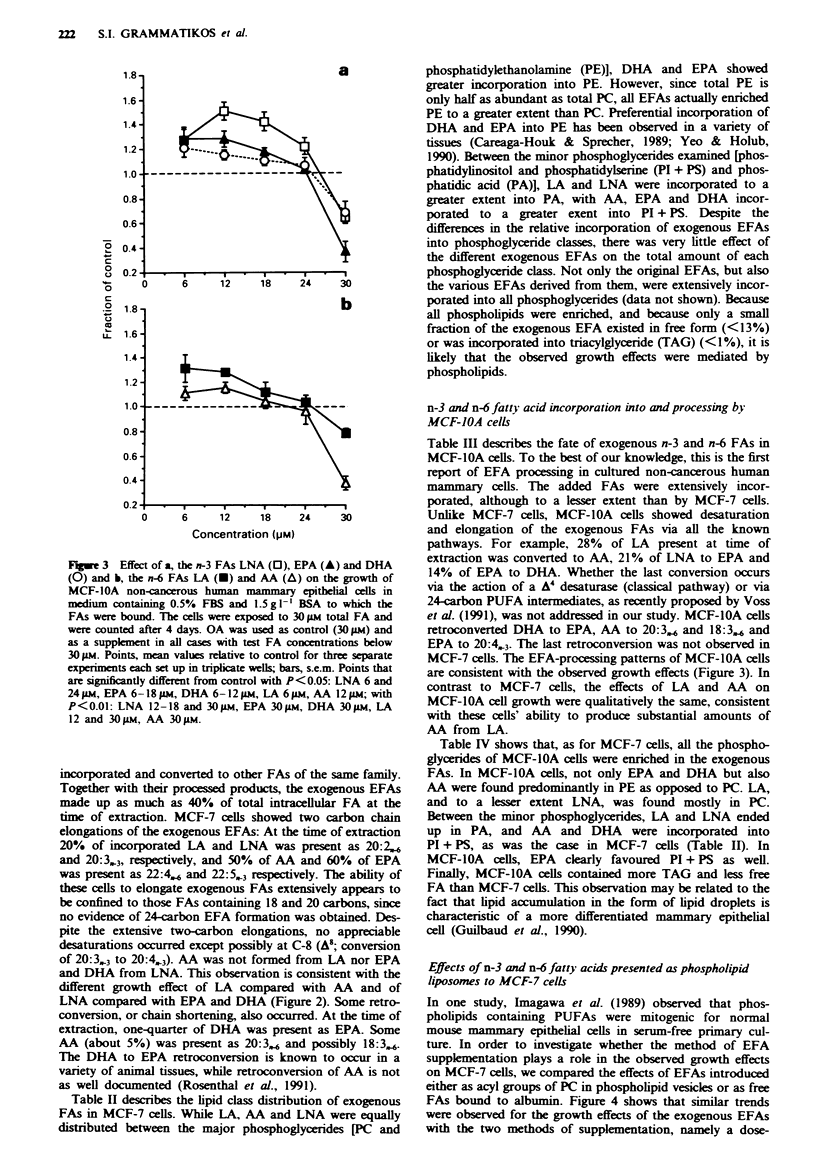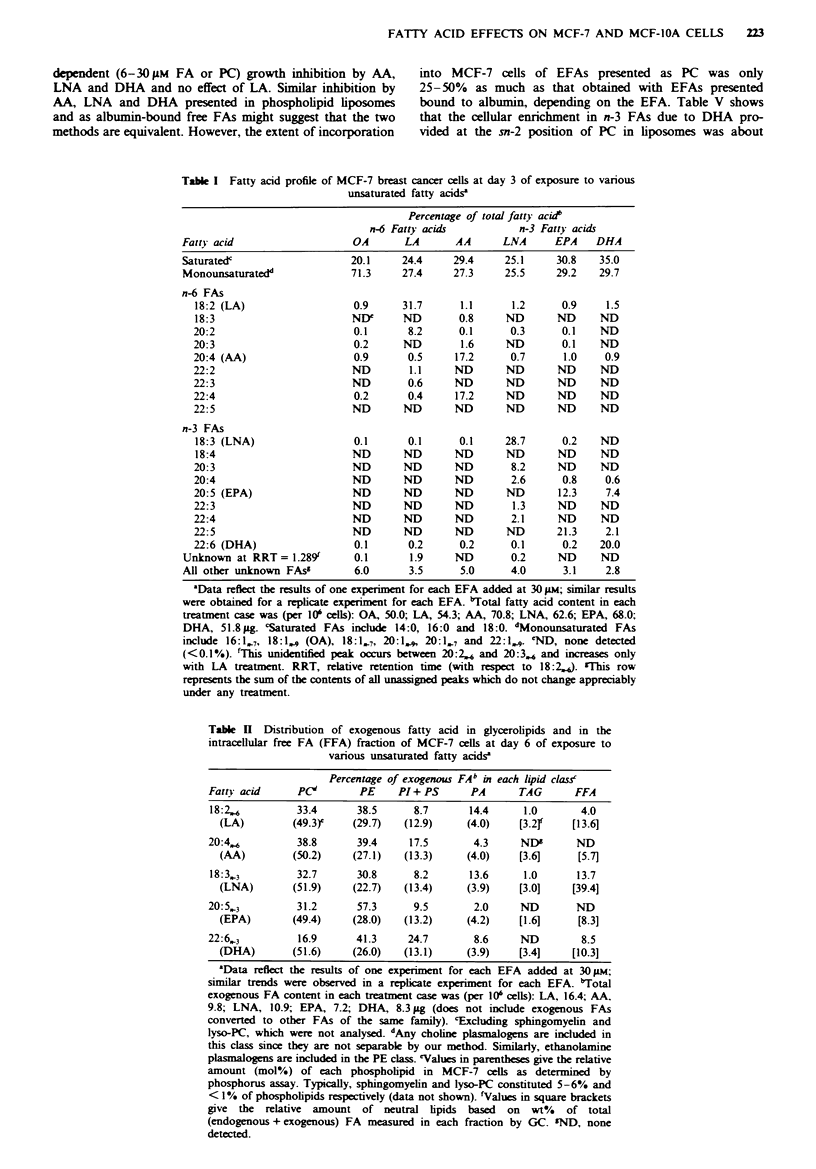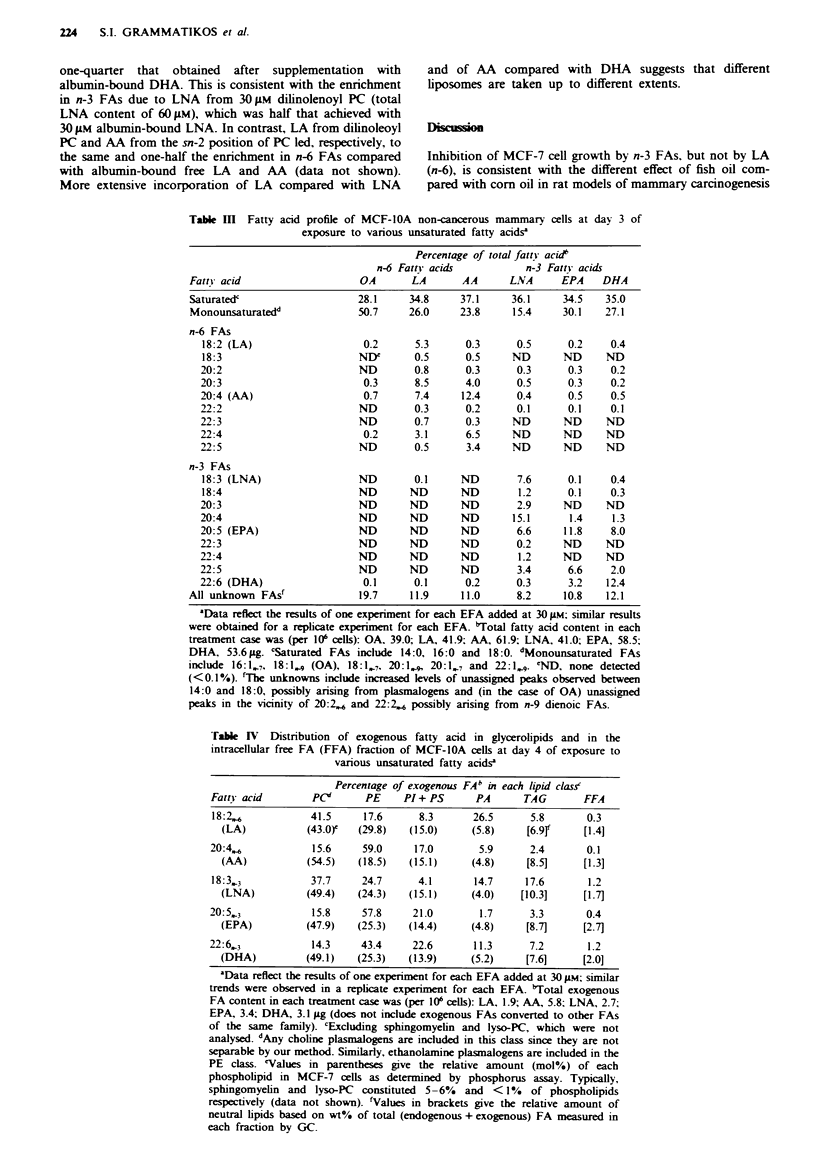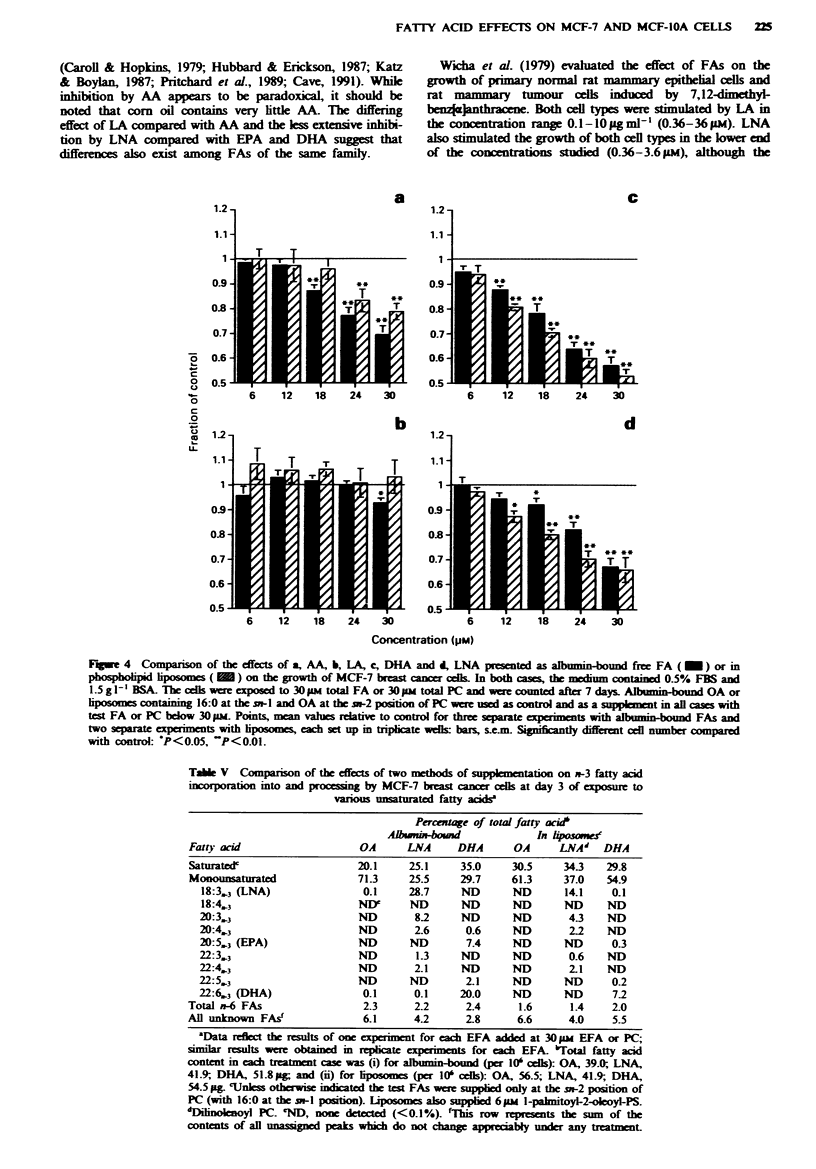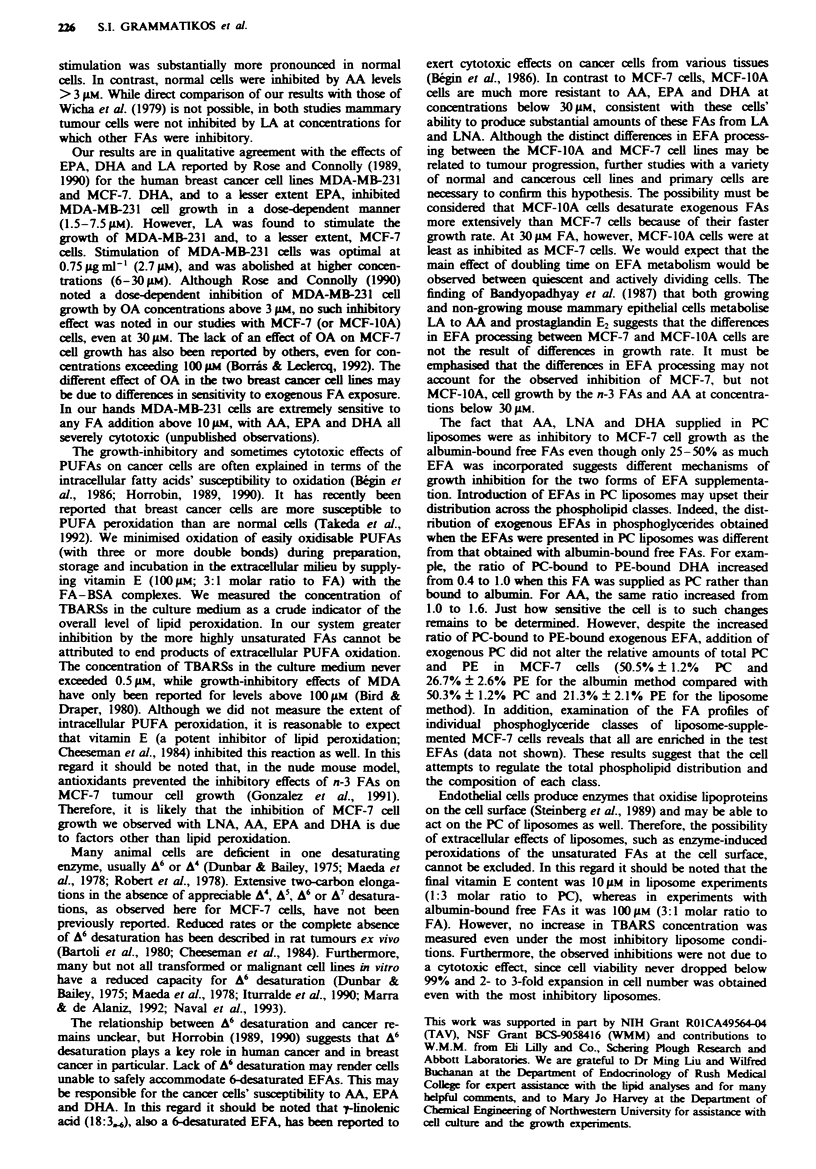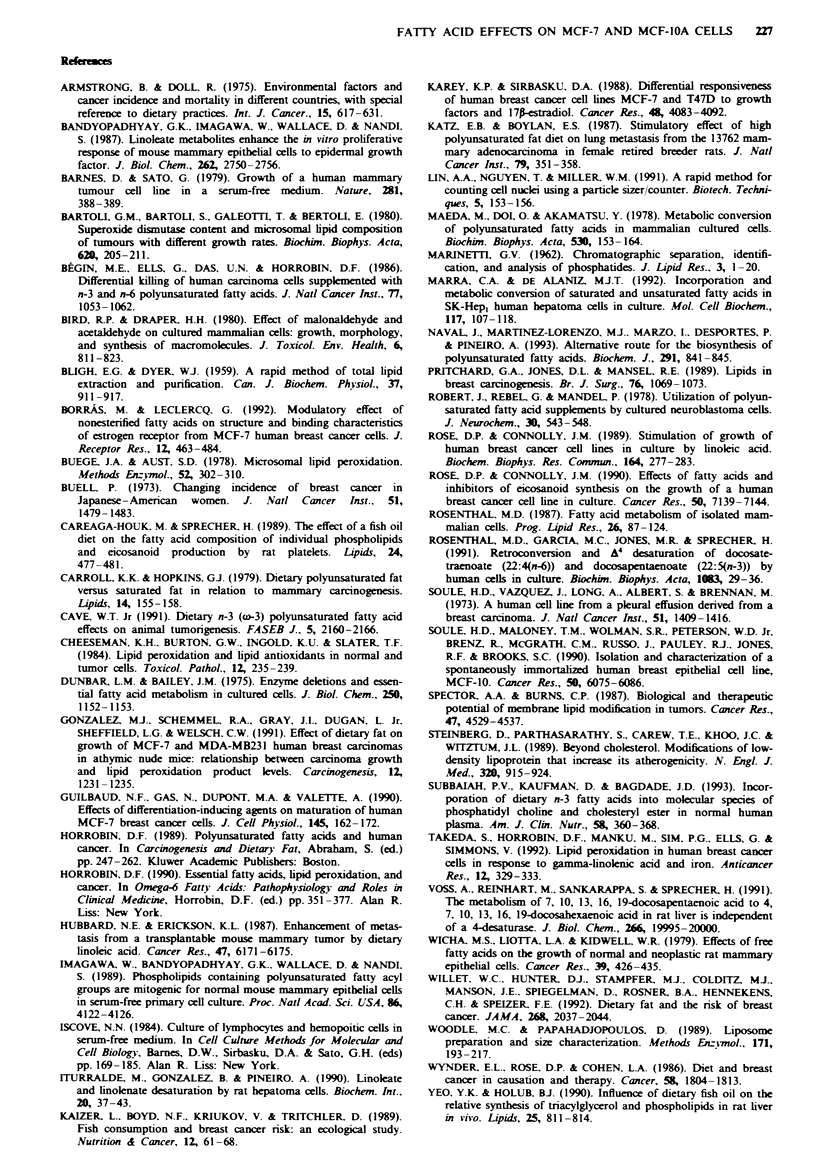# n-3 and n-6 fatty acid processing and growth effects in neoplastic and non-cancerous human mammary epithelial cell lines.

**DOI:** 10.1038/bjc.1994.283

**Published:** 1994-08

**Authors:** S. I. Grammatikos, P. V. Subbaiah, T. A. Victor, W. M. Miller

**Affiliations:** Department of Chemical Engineering, Northwestern University, Evanston, Illinois 60208-3120.

## Abstract

The type rather than the amount of dietary fat may be more important in breast carcinogenesis. While animal studies support this view, little is known about the effects of essential fatty acids (EFAs) at the cellular level. The MCF-7 breast cancer and the MCF-10A non-cancerous human mammary epithelial cell lines are compared in terms of growth response to EFAs and ability to incorporate and process the EFAs. Eicosapentaenoic (EPA, n-3) and docosahexaenoic (DHA, n-3) acids, presented bound to albumin, inhibited the growth of MCF-7 cells by as much as 50% in a dose-dependent manner (6-30 microM) in medium containing 0.5% serum. alpha-Linolenic (LNA, n-3) and arachidonic (AA, n-6) acids inhibited growth less extensively, while linoleic acid (LA, n-6) had no effect. In contrast, MCF-10A cells were not inhibited by any of the EFAs at levels below 24 microM. The differential effects of AA, EPA and DHA on MCF-7 and MCF-10A cells support a protective role of highly unsaturated essential fatty acids against breast cancer. The EFAs were primarily incorporated into phosphoglycerides. MCF-7 cells showed chain elongations and possibly delta 8 desaturation, but no AA was formed from LA, nor EPA or DHA from LNA. In contrast, MCF-10A cells desaturated and elongated the exogenous EFAs via all the known pathways. These findings suggest defects in the desaturating enzymes of MCF-7 cells. LNA, DHA and AA presented to MCF-7 cells in phospholipid liposomes inhibited growth as extensively as albumin-bound free acids, but were less extensively incorporated, suggesting different mechanisms of inhibition for the two methods.


Br. J. Cancer (1994), 70, 219-227 © Macmillan Press Ltd., 1994

### n-3 and n-6 fatty acid processing and growth effects in neoplastic and non-cancerous human mammary epithelial cell lines

S.I. Grammatikos[1], P.V. Subbaiah[2], T.A. Victor[3] & W.M. Miller[1]

[1]Department of Chemical Engineering, Northwestern University, Evanston, Illinois 60208-3120, USA; [2]Departments of Biochemistry and Medicine, Rush Medical College, Chicago, Illinois 60612, USA; [3]Department of Pathology, Northwestern University Medical School, Evanston Hospital, Evanston, Illinois 60201, USA.

**Summary** The type rather than the amount of dietary fat may be more important in breast carcinogenesis. While animal studies support this view, little is known about the effects of essential fatty acids (EFAs) at the cellular level. The MCF-7 breast cancer and the MCF-10A non-cancerous human mammary epithelial cell lines are compared in terms of growth response to EFAs and ability to incorporate and process the EFAs. Eicosapentaenoic (EPA, n-3) and docosahexaenoic (DHA, n-3) acids, presented bound to albumin, inhibited the growth of MCF-7 cells by as much as 50% in a dose-dependent manner (6-30 μM) in medium containing 0.5% serum. α-Linolenic (LNA, n-3) and arachidonic (AA, n-6) acids inhibited growth less extensively, while linoleic acid (LA, n-6) had no effect. In contrast, MCF-10A cells were not inhibited by any of the EFAs at levels below 24 μM. The differential effects of AA, EPA and DHA on MCF-7 and MCF-10A cells support a protective role of highly unsaturated essential fatty acids against breast cancer. The EFAs were primarily incorporated into phosphoglycerides. MCF-7 cells showed chain elongations and possibly $\Delta^8$ desaturation, but no AA was formed from LA, nor EPA or DHA from LNA. In contrast, MCF-10A cells desaturated and elongated the exogenous EFAs via all the known pathways. These findings suggest defects in the desaturating enzymes of MCF-7 cells. LNA, DHA and AA presented to MCF-7 cells in phospholipid liposomes inhibited growth as extensively as albumin-bound free acids, but were less extensively incorporated, suggesting different mechanisms of inhibition for the two methods.

Differences in the rates of breast cancer incidence among women in different countries and corresponding changes in the incidence of breast cancer for women who migrate from an area of lower incidence to one of higher incidence suggest that environmental factors such as dietary fat may play a role in this disease (Buell, 1978; Armstrong & Doll, 1975). Epidemiological studies testing this hypothesis have produced conflicting results (Wynder et al., 1986; Willet et al., 1992). The apparent lack of correlation between total dietary fat intake and the incidence of breast cancer led to the idea that the type of fatty acid (FA) in the diet might play a more important role in carcinogenesis than total dietary fat (Cave, 1991). The type of FA can have a direct effect because, unlike proteins and carbohydrates, FAs are incorporated directly into membranes. In particular, the essential FAs (n-3 and n-6, EFAs), which mammals cannot synthesise, are either incorporated intact or converted to other FAs of the same family. Experiments in rat models showed that diets rich in linoleic acid ($18:2_{n-6}$, LA) increased the incidence and metastasis of chemically induced and transplanted mammary tumours (Carroll & Hopkins, 1979; Hubbard & Erickson, 1987; Katz & Boylan, 1987). Conversely, diets rich in n-3 FAs, such as α-linolenic (18:3, LNA), eicosapentaenoic (20:5, EPA) and docosahexaenoic (22:6, DHA) acids, reduced the incidence, growth and metastasis of both induced and transplanted rat mammary tumours (Pritchard et al., 1989; Cave, 1991). These results could explain the increasing incidence of breast cancer in the USA, where LA-rich vegetable oil consumption has steadily increased, as well as the lower incidence in countries where fish oils (rich in n-3 FAs) constitute a higher proportion of the dietary lipid intake (Carroll & Hopkins, 1979; Kaizer et al., 1989).

The effects of n-6 and n-3 FAs on mammary carcinogenesis may be direct, involving the mammary cells at any step in the carcinogenic process, or may be mediated by accessory cells or organs. Possible mechanisms include increased peroxidation of FAs with a high degree of unsaturation, modification of membrane structure and function and modulation of signal transduction, eicosanoid hormone levels and gene expression. Since the membrane FA composition of cells in culture can be modified without changing the cholesterol, phospholipid or protein content (Spector & Burns, 1987), these mechanisms can be investigated *in vitro* at the cellular level. To date, there have been very few attempts to study the effects of dietary FAs on breast cancer at the cellular level (Wicha et al., 1979; Rose & Connolly, 1990).

The intracellular fate of exogenous EFAs is of central importance to any study of the effect of dietary fat at the cellular level. The EFAs can theoretically undergo stepwise two-carbon chain elongations or shortenings, as well as desaturations or saturations, leading to a variety of FAs of the same family (Figure 1). Although processing of this type is believed to occur primarily in the liver, the desaturating and elongating ability of a variety of primary cell cultures and cell lines has been demonstrated (reviewed by Rosenthal, 1987). While many transformed or malignant cells have limited capacity to perform FA desaturations (Dunbar & Bailey, 1975; Iturralde et al., 1990; Marra & de Alaniz, 1992; Naval et al., 1993), very little is known about the ability of cancerous and normal human mammary epithelial cells to elongate and/or desaturate exogenous FAs in culture.

In this report we compare the MCF-7 human breast cancer cell line (Soule et al., 1973) with the recently established non-cancerous human mammary epithelial cell line MCF-10A (Soule et al., 1990) in terms of growth response to the n-6 FAs LA and AA, and to the n-3 FAs LNA, EPA and DHA. We also compare their ability to incorporate, elongate and desaturate these FAs. Our findings indicate important differences between the two cell lines and set the stage for an evaluation of dietary fatty acid effects on cultured human mammary cells from all stages of the carcinogenic process.

### Materials and methods

### Materials

Milli-Q water (Millipore) was used throughout. Stabilised bovine fibronectin was purchased from Biomedical Technologies (Stoughton, MA, USA); Miles Pentex bovine serum albumin (BSA, fraction V, fatty acid-free, very low

Correspondence: W.M. Miller, Department of Chemical Engineering, Northwestern University, 2145 Sheridan Road, Evanston, Illinois 60208-3120, USA.
Received 22 December 1993; and in revised form 6 April 1994.

**Figure 1** Metabolism of the parent essential fatty acids linoleic and α-linolenic. Various eicosanoid hormones are produced from the 20-carbon FAs containing at least three double bonds.

endotoxin) from The Binding Site (San Diego, CA, USA); all phospholipids except those used as thin-layer chromatography (TLC) standards from Avanti Polar Lipids (Alabaster, AL, USA); fetal bovine serum (FBS, lot no. 91103) from Bioproducts For Science (Indianapolis, IN, USA); L-glutamine and trypsin-EDTA from Biologos (Naperville, IL, USA); penicillin-streptomycin from Gibco; solvents from American Burdick & Jackson (Muskegon, MI, USA); glacial acetic acid from Fischer Scientific; 70% perchloric acid from Mallinkrodt and $BF_3$/methanol (14%, w/v) from Alltech. All other chemicals, media, and supplements were purchased from Sigma.

### *Cell culture*

MCF-7 and MCF-10A cells were purchased from the American Type Culture Collection (Rockville, MD, USA) at passages 148 and 40 respectively. To minimise interference from serum FAs and to obtain a better defined system, all experiments employed cells adapted for growth in low-serum medium (LSM): A 1:1 (v/v) mixture of Dulbecco's modified Eagle medium (DMEM) (Sigma D-5648) and Ham's F-12 (Sigma N-6760) supplemented with 15 mM HEPES, 25 mM sodium bicarbonate, L-glutamine (to 6 mM), 30 nM sodium selenite, 25 mg $l^{-1}$ human transferrin, 10 mg $l^{-1}$ bovine insulin, 10 nM 17β-oestradiol, 50 μg $l^{-1}$ epidermal growth factor (human, recombinant), 2 mg $l^{-1}$ hydrocortisone, 20 μg $l^{-1}$ 3,3′,5-triiodo-L-thyronine, 50 μg $l^{-1}$ cholera toxin, 0.2 mM ethanolamine, 1.5 g $l^{-1}$ fatty acid-free BSA (final concentration in all cultures), 0.5 ml $l^{-1}$ bovine serum lipids (Sigma L-4646) and 0.5% (v/v) FBS, pH 7.4. Although LSM contains components necessary for MCF-7 cell growth in the absence of serum (Barnes & Sato, 1979; Karey & Sirbasku, 1988), it does not support MCF-7 cell growth at FBS levels below 0.5%. MCF-7 cells grow more slowly in LSM than do MCF-10A cells (doubling time ~48 h *vs* 24 h). Cells were routinely cultured in LSM without antibiotics, and were passaged weekly at 80-100% confluency with trypsin-EDTA (inactivated using soybean trypsin inhibitor). In all FA experiments LSM was used without bovine serum lipids (LSM/$L^-$), and was supplemented with antibiotics. All culture surfaces were coated prior to use with 1 μg $cm^{-2}$ bovine fibronectin (15 μg $ml^{-1}$ in phosphate-buffered saline, pH 7.4).

### *Growth experiments*

MCF-7 cells (at passages 182-200) and MCF-10A cells (at passages 50-60) in LSM were harvested from stock cultures in exponential growth, and passed twice through a 22 G needle to obtain single cells. Approximately $7 \times 10^4$ MCF-7 cells per well ($4 \times 10^4$ MCF-10A cells per well) were cultured in six-well plates (Corning) for 48 h (24 h for MCF-10A cells) in LSM/$L^-$. The FAs were then introduced either bound to BSA or in phosphatidylcholine (PC) liposomes, and incubation continued for 7 days (4 days for MCF-10A cells). These seeding densities and incubation times resulted in similar final cell densities of control cells and similar variation in medium pH (final pH 7.0-7.1 without feeding) in all experiments with both cell lines. In all cases the cells were exposed to 30 μM total FA or 30 μM total PC. BSA-bound oleic acid (18:$1_{n-9}$, OA) (30 μM) or liposomes containing palmitic acid (16:0) at the *sn*-1 and OA at the *sn*-2 position of PC (30 μM) were used as control and as a supplement in all cases with test FA or PC below 30 μM. OA was used as control because it is a non-essential monounsaturated FA abundant in cell membranes, and at 30 μM it did not affect the growth of either MCF-7 or MCF-10A cells as compared with growth in LSM. Cell numbers were determined by nuclei counts as described by Lin *et al.* (1991), except that cells removed from each well with 0.5 ml of trypsin-EDTA were combined with 0.5 ml of LSM/$L^-$ with 5% (v/v) FBS and 1 ml of 2 × hypotonic solution (40 g $l^{-1}$ Triton X-100, 0.2 M citric acid). The effect of the highest levels of exogenous lipids on cell viability was assessed by trypan blue exclusion using cells from plates set up in parallel with the growth experiments.

### *Fatty acid binding to albumin and liposome preparation*

Cholesterol (in chloroform) and FA (5 mg) and vitamin E (in ethanol) were sequentially added to a test tube with solvent evaporation. BSA solution (400 g $l^{-1}$) was added, and the mixture was sonicated under nitrogen using a bath sonicator, adjusted to pH 7.4, diluted to 2.5 ml, filtered and stored at 4°C under argon. Addition of 30 μM total FA to the cultures provided 1 g $l^{-1}$ BSA, 12 μM cholesterol and 100 μM vitamin E.

Vesicles consisting primarily of PC, with the FA of interest either at the *sn*-2 position with 16:0 at the *sn*-1 position (for OA, AA and DHA) or at both the *sn*-1 and *sn*-2 positions (for LA and LNA), were prepared by probe-tip sonication following the guidelines of Woodle and Papahadjopoulos (1989) and Iscove (1984). The liposomes contained 1-palmitoyl-2-oleoyl-phosphatidylserine (PS), cholesterol and vitamin E at 1:5, 1:1 and 1:3 molar ratios to PC respectively. Filtered (0.22 μm) liposome suspensions were stored at 4°C under argon and used within 2 weeks of preparation. Quantification by phosphorus assay and gas chromatographic (GC) analysis confirmed that no appreciable loss of material occurred during liposome or FA-BSA preparation.

### *Cell culture and extraction for lipid analysis*

Cultures were initiated as for the growth experiments described above, except that 75 $cm^2$ T flasks were used with $1.5-2.0 \times 10^6$ MCF-7 cells per flask or $1.0 \times 10^6$ MCF-10A cells per flask. Only the highest level (30 μM FA or 30 μM PC) of each FA was studied. Total lipid extracts were prepared at days 3 and 6 (days 3 and 4 for MCF-10A cells) after FA addition using the method of Bligh and Dyer (1959), and contained 5 μg of heptadecanoic acid (17:0, HDA) as internal standard. Day 3 was chosen for total lipid analysis because cultured cells are known to take up exogenous FAs quite rapidly, and FA uptake and metabolism undoubtedly precede any FA-induced growth effects. Analysis of various glycerolipid fractions was routinely performed at day 6 (day 4 for MCF-10A cells). No discrepancies between FA profiles at days 3 and 6 (or 4) were noted.

*Thin-layer chromatography (TLC)*

Prior to TLC, extracts were dried under nitrogen and redissolved in chloroform containing 0.1 g $l^{-1}$ butylated hydroxytoluene. Aliquots were loaded onto silica gel TLC plates, using chloroform–methanol–acetic acid–0.15 M sodium chloride (50:25:8:2.5, v/v) to separate and quantify the phospholipid classes. The remaining material was fractionated in a two-step TLC system [first step as above; second step in hexane–diethyl ether–acetic acid (70:30:1, v/v)], which also separates the neutral lipid classes. The spots were identified by parallel analysis of phospholipid and neutral lipid standards, and analysed for FA content by GC.

*Thiobarbituric acid-reactive substances and phosphorus assays*

The extent of exogenous lipid peroxidation was characterised by the level of thiobarbituric acid-reactive substances (TBARSs), primarily malondialdehyde (MDA), measured in spent culture medium using the method of Buege and Aust (1978). The modified Bartlett procedure for total phosphorus (Martinetti, 1962) was used for quantification of liposome preparations and phospholipid classes separated by TLC.

*Gas chromatography (GC)*

Extracts were dried under nitrogen and redissolved in 1 ml of GC-grade petroleum ether. FA methyl esters were prepared using $BF_3$/methanol (14%, w/v), and analysed by capillary GC as previously described (Subbaiah *et al.*, 1993). Phospholipid and neutral lipid classes scraped from TLC plates were treated directly with $BF_3$/methanol and supplemented with 1 μg of HDA.

*Statistical analyses*

Statistical significance of the growth results was assessed by paired Student's *t*-test on the raw data from all experiments (three experiments each in triplicate for each albumin-bound FA; two experiments each in triplicate for each FA presented in PC liposomes).

### Results

*Effects of n-3 and n-6 fatty acids on cell growth*

*MCF-7 cells* We examined the influence of three *n*-3 (LNA, EPA and DHA) and two *n*-6 (LA and AA) albumin-bound FAs on the growth of MCF-7 cells (Figure 2). All *n*-3 FAs tested inhibited cell growth in a dose-dependent manner (6–30 μM), with EPA and DHA being most effective. For the *n*-6 FAs, LA had no effect over the same concentration range, while AA was as inhibitory as LNA. Inhibition by even the highest levels of FAs tested was not due to a cytotoxic effect since the cell number increased to at least twice the seeding density, no appreciable cell detachment was observed during the culture period and the viability of attached cells at the end of each experiment never dropped below 99%. Greater inhibition by the more highly unsaturated FAs cannot be attributed to end products of polyunsaturated FA (PUFA) oxidation in the medium. In these and similar experiments (for measurement of FA incorporation and processing), the concentration of TBARSs in the culture medium never exceeded 0.5 μM at days 3, 6 and 7 of culture (results not shown).

*MCF-10A cells* MCF-10A cells were not inhibited by any of the albumin-bound *n*-3 or *n*-6 FAs at levels below 24 μM, and were even stimulated by as much as 50% at the lower cencentrations (Figure 3). At 30 μM FA, however, the cells were dramatically inhibited by AA and EPA, and moderately inhibited by the remaining FAs. It is interesting to note that MCF-10A cells were more extensively inhibited by 30 μM AA and EPA than were MCF-7 cells. As was the case with

**Figure 2** Effect of **a**, the *n*-3 FAs LNA (□), EPA (▲) and DHA (○) and **b**, the *n*-6 FAs LA (■) and AA (△) on the growth of MCF-7 breast cancer cells in medium containing 0.5% FBS and 1.5 g $l^{-1}$ BSA to which the FAs were bound. The cells were exposed to 30 μM total FA and were counted after 7 days. OA was used as control (30 μM) and as a supplement in all cases with test FA concentrations below 30 μM. Points, mean values relative to control for three separate experiments, each set up in triplicate wells; bars, s.e.m. Points that are significantly different from control with $P<0.05$: LNA 18 μM, EPA 6 μM, LA 30 μM; with $P<0.01$: LNA 24–30 μM, EPA 12–30 μM, DHA 12–30 μM, AA 18–30 μM.

MCF-7 cells, growth inhibition was not due to a cytotoxic effect, and the concentration of extracellular TBARSs never exceeded 0.5 μM. The differences between MCF-7 and MCF-10A cell growth responses to the exogenous EFAs are not due to the different growth rates of the two cell lines because faster growing MCF-7 cells cultured in medium containing 5% FBS were also inhibited by AA and DHA, but not by LA. Low-serum-containing medium was used to minimise interference from serum FAs and to enable evaluation of FA incorporation and processing without the need for radiolabelled compounds.

*n-3 and n-6 fatty acid incorporation into and processing by MCF-7 cells*

MCF-7 cells cultured in medium containing 5% FBS without any other FA additions were found to contain primarily monounsaturated FAs (54% of total intracellular FA, mainly oleic and palmitoleic acids) and saturated FAs (37%). Essential FAs (mainly LA and AA) obtained from serum constituted 9% of total FA.

In order to define the EFA processing patterns of MCF-7 cells and to explain the different growth effects of LA and AA, we measured the extent of EFA incorporation into and processing by these cells in the same low-serum-containing medium used in the growth experiments described above ($LSM/L^-$). Table I shows that the exogenous FAs were

**Figure 3** Effect of **a**, the *n*-3 FAs LNA (□), EPA (▲) and DHA (○) and **b**, the *n*-6 FAs LA (■) and AA (△) on the growth of MCF-10A non-cancerous human mammary epithelial cells in medium containing 0.5% FBS and $1.5\,g\,l^{-1}$ BSA to which the FAs were bound. The cells were exposed to 30 μM total FA and were counted after 4 days. OA was used as control (30 μM) and as a supplement in all cases with test FA concentrations below 30 μM. Points, mean values relative to control for three separate experiments each set up in triplicate wells; bars, s.e.m. Points that are significantly different from control with $P<0.05$: LNA 6 and 24 μM, EPA 6–18 μM, DHA 6–12 μM, LA 6 μM, AA 12 μM; with $P<0.01$: LNA 12–18 and 30 μM, EPA 30 μM, DHA 30 μM, LA 12 and 30 μM, AA 30 μM.

incorporated and converted to other FAs of the same family. Together with their processed products, the exogenous EFAs made up as much as 40% of total intracellular FA at the time of extraction. MCF-7 cells showed two carbon chain elongations of the exogenous EFAs: At the time of extraction 20% of incorporated LA and LNA was present as $20{:}2_{n6}$ and $20{:}3_{n3}$, respectively, and 50% of AA and 60% of EPA was present as $22{:}4_{n6}$ and $22{:}5_{n3}$ respectively. The ability of these cells to elongate exogenous FAs extensively appears to be confined to those FAs containing 18 and 20 carbons, since no evidence of 24-carbon EFA formation was obtained. Despite the extensive two-carbon elongations, no appreciable desaturations occurred except possibly at C-8 ($\Delta^8$; conversion of $20{:}3_{n3}$ to $20{:}4_{n3}$). AA was not formed from LA nor EPA and DHA from LNA. This observation is consistent with the different growth effect of LA compared with AA and of LNA compared with EPA and DHA (Figure 2). Some retroconversion, or chain shortening, also occurred. At the time of extraction, one-quarter of DHA was present as EPA. Some AA (about 5%) was present as $20{:}3_{n6}$ and possibly $18{:}3_{n6}$. The DHA to EPA retroconversion is known to occur in a variety of animal tissues, while retroconversion of AA is not as well documented (Rosenthal *et al.*, 1991).

Table II describes the lipid class distribution of exogenous FAs in MCF-7 cells. While LA, AA and LNA were equally distributed between the major phosphoglycerides [PC and phosphatidylethanolamine (PE)], DHA and EPA showed greater incorporation into PE. However, since total PE is only half as abundant as total PC, all EFAs actually enriched PE to a greater extent than PC. Preferential incorporation of DHA and EPA into PE has been observed in a variety of tissues (Careaga-Houk & Sprecher, 1989; Yeo & Holub, 1990). Between the minor phosphoglycerides examined [phosphatidylinositol and phosphatidylserine (PI + PS) and phosphatidic acid (PA)], LA and LNA were incorporated to a greater extent into PA, with AA, EPA and DHA incorporated to a greater exent into PI + PS. Despite the differences in the relative incorporation of exogenous EFAs into phosphoglyceride classes, there was very little effect of the different exogenous EFAs on the total amount of each phosphoglyceride class. Not only the original EFAs, but also the various EFAs derived from them, were extensively incorporated into all phosphoglycerides (data not shown). Because all phospholipids were enriched, and because only a small fraction of the exogenous EFA existed in free form (<13%) or was incorporated into triacylglyceride (TAG) (<1%), it is likely that the observed growth effects were mediated by phospholipids.

### *n-3 and n-6 fatty acid incorporation into and processing by MCF-10A cells*

Table III describes the fate of exogenous *n*-3 and *n*-6 FAs in MCF-10A cells. To the best of our knowledge, this is the first report of EFA processing in cultured non-cancerous human mammary cells. The added FAs were extensively incorporated, although to a lesser extent than by MCF-7 cells. Unlike MCF-7 cells, MCF-10A cells showed desaturation and elongation of the exogenous FAs via all the known pathways. For example, 28% of LA present at time of extraction was converted to AA, 21% of LNA to EPA and 14% of EPA to DHA. Whether the last conversion occurs via the action of a $\Delta^4$ desaturase (classical pathway) or via 24-carbon PUFA intermediates, as recently proposed by Voss *et al.* (1991), was not addressed in our study. MCF-10A cells retroconverted DHA to EPA, AA to $20{:}3_{n6}$ and $18{:}3_{n6}$ and EPA to $20{:}4_{n3}$. The last retroconversion was not observed in MCF-7 cells. The EFA-processing patterns of MCF-10A cells are consistent with the observed growth effects (Figure 3). In contrast to MCF-7 cells, the effects of LA and AA on MCF-10A cell growth were qualitatively the same, consistent with these cells' ability to produce substantial amounts of AA from LA.

Table IV shows that, as for MCF-7 cells, all the phosphoglycerides of MCF-10A cells were enriched in the exogenous FAs. In MCF-10A cells, not only EPA and DHA but also AA were found predominantly in PE as opposed to PC. LA, and to a lesser extent LNA, was found mostly in PC. Between the minor phosphoglycerides, LA and LNA ended up in PA, and AA and DHA were incorporated into PI + PS, as was the case in MCF-7 cells (Table II). In MCF-10A cells, EPA clearly favoured PI + PS as well. Finally, MCF-10A cells contained more TAG and less free FA than MCF-7 cells. This observation may be related to the fact that lipid accumulation in the form of lipid droplets is characteristic of a more differentiated mammary epithelial cell (Guilbaud *et al.*, 1990).

### *Effects of n-3 and n-6 fatty acids presented as phospholipid liposomes to MCF-7 cells*

In one study, Imagawa *et al.* (1989) observed that phospholipids containing PUFAs were mitogenic for normal mouse mammary epithelial cells in serum-free primary culture. In order to investigate whether the method of EFA supplementation plays a role in the observed growth effects on MCF-7 cells, we compared the effects of EFAs introduced either as acyl groups of PC in phospholipid vesicles or as free FAs bound to albumin. Figure 4 shows that similar trends were observed for the growth effects of the exogenous EFAs with the two methods of supplementation, namely a dosedependent (6–30 μM FA or PC) growth inhibition by AA, LNA and DHA and no effect of LA. Similar inhibition by AA, LNA and DHA presented in phospholipid liposomes and as albumin-bound free FAs might suggest that the two methods are equivalent. However, the extent of incorporation into MCF-7 cells of EFAs presented as PC was only 25–50% as much as that obtained with EFAs presented bound to albumin, depending on the EFA. Table V shows that the cellular enrichment in *n*-3 FAs due to DHA provided at the *sn*-2 position of PC in liposomes was about

**Table I** Fatty acid profile of MCF-7 breast cancer cells at day 3 of exposure to various unsaturated fatty acids[a]

| | Percentage of total fatty acid[b] | | | | | |
|---|---|---|---|---|---|---|
| | | *n-6 Fatty acids* | | | *n-3 Fatty acids* | |
| *Fatty acid* | *OA* | *LA* | *AA* | *LNA* | *EPA* | *DHA* |
| Saturated[c] | 20.1 | 24.4 | 29.4 | 25.1 | 30.8 | 35.0 |
| Monounsaturated[d] | 71.3 | 27.4 | 27.3 | 25.5 | 29.2 | 29.7 |
| *n*-6 FAs | | | | | | |
| 18:2 (LA) | 0.9 | 31.7 | 1.1 | 1.2 | 0.9 | 1.5 |
| 18:3 | ND[e] | ND | 0.8 | ND | ND | ND |
| 20:2 | 0.1 | 8.2 | 0.1 | 0.3 | 0.1 | ND |
| 20:3 | 0.2 | ND | 1.6 | ND | 0.1 | ND |
| 20:4 (AA) | 0.9 | 0.5 | 17.2 | 0.7 | 1.0 | 0.9 |
| 22:2 | ND | 1.1 | ND | ND | ND | ND |
| 22:3 | ND | 0.6 | ND | ND | ND | ND |
| 22:4 | 0.2 | 0.4 | 17.2 | ND | ND | ND |
| 22:5 | ND | ND | ND | ND | ND | ND |
| *n*-3 FAs | | | | | | |
| 18:3 (LNA) | 0.1 | 0.1 | 0.1 | 28.7 | 0.2 | ND |
| 18:4 | ND | ND | ND | ND | ND | ND |
| 20:3 | ND | ND | ND | 8.2 | ND | ND |
| 20:4 | ND | ND | ND | 2.6 | 0.8 | 0.6 |
| 20:5 (EPA) | ND | ND | ND | ND | 12.3 | 7.4 |
| 22:3 | ND | ND | ND | 1.3 | ND | ND |
| 22:4 | ND | ND | ND | 2.1 | ND | ND |
| 22:5 | ND | ND | ND | ND | 21.3 | 2.1 |
| 22:6 (DHA) | 0.1 | 0.2 | 0.2 | 0.1 | 0.2 | 20.0 |
| Unknown at RRT = 1.289[f] | 0.1 | 1.9 | ND | 0.2 | ND | ND |
| All other unknown FAs[g] | 6.0 | 3.5 | 5.0 | 4.0 | 3.1 | 2.8 |

[a]Data reflect the results of one experiment for each EFA added at 30 μM; similar results were obtained for a replicate experiment for each EFA. [b]Total fatty acid content in each treatment case was (per $10^6$ cells): OA, 50.0; LA, 54.3; AA, 70.8; LNA, 62.6; EPA, 68.0; DHA, 51.8 μg. [c]Saturated FAs include 14:0, 16:0 and 18:0. [d]Monounsaturated FAs include $16{:}1_{n-7}$, $18{:}1_{n-9}$ (OA), $18{:}1_{n-7}$, $20{:}1_{n-9}$, $20{:}1_{n-7}$ and $22{:}1_{n-9}$. [e]ND, none detected (<0.1%). [f]This unidentified peak occurs between $20{:}2_{n-6}$ and $20{:}3_{n-6}$ and increases only with LA treatment. RRT, relative retention time (with respect to $18{:}2_{n-6}$). [g]This row represents the sum of the contents of all unassigned peaks which do not change appreciably under any treatment.

**Table II** Distribution of exogenous fatty acid in glycerolipids and in the intracellular free FA (FFA) fraction of MCF-7 cells at day 6 of exposure to various unsaturated fatty acids[a]

| | *Percentage of exogenous FA[b] in each lipid class[c]* | | | | | |
|---|---|---|---|---|---|---|
| *Fatty acid* | *PC[d]* | *PE* | *PI + PS* | *PA* | *TAG* | *FFA* |
| $18{:}2_{n-6}$ | 33.4 | 38.5 | 8.7 | 14.4 | 1.0 | 4.0 |
| (LA) | (49.3)[e] | (29.7) | (12.9) | (4.0) | [3.2][f] | [13.6] |
| $20{:}4_{n-6}$ | 38.8 | 39.4 | 17.5 | 4.3 | ND[g] | ND |
| (AA) | (50.2) | (27.1) | (13.3) | (4.0) | [3.6] | [5.7] |
| $18{:}3_{n-3}$ | 32.7 | 30.8 | 8.2 | 13.6 | 1.0 | 13.7 |
| (LNA) | (51.9) | (22.7) | (13.4) | (3.9) | [3.0] | [39.4] |
| $20{:}5_{n-3}$ | 31.2 | 57.3 | 9.5 | 2.0 | ND | ND |
| (EPA) | (49.4) | (28.0) | (13.2) | (4.2) | [1.6] | [8.3] |
| $22{:}6_{n-3}$ | 16.9 | 41.3 | 24.7 | 8.6 | ND | 8.5 |
| (DHA) | (51.6) | (26.0) | (13.1) | (3.9) | [3.4] | [10.3] |

[a]Data reflect the results of one experiment for each EFA added at 30 μM; similar trends were observed in a replicate experiment for each EFA. [b]Total exogenous FA content in each treatment case was (per $10^6$ cells): LA, 16.4; AA, 9.8; LNA, 10.9; EPA, 7.2; DHA, 8.3 μg (does not include exogenous FAs converted to other FAs of the same family). [c]Excluding sphingomyelin and lyso-PC, which were not analysed. [d]Any choline plasmalogens are included in this class since they are not separable by our method. Similarly, ethanolamine plasmalogens are included in the PE class. [e]Values in parentheses give the relative amount (mol%) of each phospholipid in MCF-7 cells as determined by phosphorus assay. Typically, sphingomyelin and lyso-PC constituted 5–6% and <1% of phospholipids respectively (data not shown). [f]Values in square brackets give the relative amount of neutral lipids based on wt% of total (endogenous + exogenous) FA measured in each fraction by GC. [g]ND, none detected.

one-quarter that obtained after supplementation with albumin-bound DHA. This is consistent with the enrichment in *n*-3 FAs due to LNA from 30 μM dilinolenoyl PC (total LNA content of 60 μM), which was half that achieved with 30 μM albumin-bound LNA. In contrast, LA from dilinoleoyl PC and AA from the *sn*-2 position of PC led, respectively, to the same and one-half the enrichment in *n*-6 FAs compared with albumin-bound free LA and AA (data not shown). More extensive incorporation of LA compared with LNA and of AA compared with DHA suggests that different liposomes are taken up to different extents.

### Discussion

Inhibition of MCF-7 cell growth by *n*-3 FAs, but not by LA (*n*-6), is consistent with the different effect of fish oil compared with corn oil in rat models of mammary carcinogenesis

**Table III** Fatty acid profile of MCF-10A non-cancerous mammary cells at day 3 of exposure to various unsaturated fatty acids[a]

| | *Percentage of total fatty acid*[b] | | | | | |
|---|---|---|---|---|---|---|
| | *n-6 Fatty acids* | | | *n-3 Fatty acids* | | |
| *Fatty acid* | *OA* | *LA* | *AA* | *LNA* | *EPA* | *DHA* |
| Saturated[c] | 28.1 | 34.8 | 37.1 | 36.1 | 34.5 | 35.0 |
| Monounsaturated[d] | 50.7 | 26.0 | 23.8 | 15.4 | 30.1 | 27.1 |
| *n*-6 FAs | | | | | | |
| 18:2 (LA) | 0.2 | 5.3 | 0.3 | 0.5 | 0.2 | 0.4 |
| 18:3 | ND[e] | 0.5 | 0.5 | ND | ND | ND |
| 20:2 | ND | 0.8 | 0.3 | 0.3 | 0.3 | 0.2 |
| 20:3 | 0.3 | 8.5 | 4.0 | 0.5 | 0.3 | 0.2 |
| 20:4 (AA) | 0.7 | 7.4 | 12.4 | 0.4 | 0.5 | 0.5 |
| 22:2 | ND | 0.3 | 0.2 | 0.1 | 0.1 | 0.1 |
| 22:3 | ND | 0.7 | 0.3 | ND | ND | ND |
| 22:4 | 0.2 | 3.1 | 6.5 | ND | ND | ND |
| 22:5 | ND | 0.5 | 3.4 | ND | ND | ND |
| *n*-3 FAs | | | | | | |
| 18:3 (LNA) | ND | 0.1 | ND | 7.6 | 0.1 | 0.4 |
| 18:4 | ND | ND | ND | 1.2 | 0.1 | 0.3 |
| 20:3 | ND | ND | ND | 2.9 | ND | ND |
| 20:4 | ND | ND | ND | 15.1 | 1.4 | 1.3 |
| 20:5 (EPA) | ND | ND | ND | 6.6 | 11.8 | 8.0 |
| 22:3 | ND | ND | ND | 0.2 | ND | ND |
| 22:4 | ND | ND | ND | 1.2 | ND | ND |
| 22:5 | ND | ND | ND | 3.4 | 6.6 | 2.0 |
| 22:6 (DHA) | 0.1 | 0.1 | 0.2 | 0.3 | 3.2 | 12.4 |
| All unknown FAs[f] | 19.7 | 11.9 | 11.0 | 8.2 | 10.8 | 12.1 |

[a]Data reflect the results of one experiment for each EFA added at 30 μM; similar results were obtained for a replicate experiment for each EFA. [b]Total fatty acid content in each treatment case was (per $10^6$ cells): OA, 39.0; LA, 41.9; AA, 61.9; LNA, 41.0; EPA, 58.5; DHA, 53.6 μg. [c]Saturated FAs include 14:0, 16:0 and 18:0. [d]Monounsaturated FAs include $16{:}1_{n-7}$, $18{:}1_{n-9}$ (OA), $18{:}1_{n-7}$, $20{:}1_{n-9}$, $20{:}1_{n-7}$ and $22{:}1_{n-9}$. [e]ND, none detected (<0.1%). [f]The unknowns include increased levels of unassigned peaks observed between 14:0 and 18:0, possibly arising from plasmalogens and (in the case of OA) unassigned peaks in the vicinity of $20{:}2_{n-6}$ and $22{:}2_{n-6}$ possibly arising from *n*-9 dienoic FAs.

**Table IV** Distribution of exogenous fatty acid in glycerolipids and in the intracellular free FA (FFA) fraction of MCF-10A cells at day 4 of exposure to various unsaturated fatty acids[a]

| | *Percentage of exogenous FA*[b] *in each lipid class*[c] | | | | | |
|---|---|---|---|---|---|---|
| *Fatty acid* | *PC*[d] | *PE* | *PI + PS* | *PA* | *TAG* | *FFA* |
| $18{:}2_{n-6}$ (LA) | 41.5 (43.0)[e] | 17.6 (29.8) | 8.3 (15.0) | 26.5 (5.8) | 5.8 [6.9][f] | 0.3 [1.4] |
| $20{:}4_{n-6}$ (AA) | 15.6 (54.5) | 59.0 (18.5) | 17.0 (15.1) | 5.9 (4.8) | 2.4 [8.5] | 0.1 [1.3] |
| $18{:}3_{n-3}$ (LNA) | 37.7 (49.4) | 24.7 (24.3) | 4.1 (15.1) | 14.7 (4.0) | 17.6 [10.3] | 1.2 [1.7] |
| $20{:}5_{n-3}$ (EPA) | 15.8 (47.9) | 57.8 (25.3) | 21.0 (14.4) | 1.7 (4.8) | 3.3 [8.7] | 0.4 [2.7] |
| $22{:}6_{n-3}$ (DHA) | 14.3 (49.1) | 43.4 (25.3) | 22.6 (13.9) | 11.3 (5.2) | 7.2 [7.6] | 1.2 [2.0] |

[a]Data reflect the results of one experiment for each EFA added at 30 μM; similar trends were observed in a replicate experiment for each EFA. [b]Total exogenous FA content in each treatment case was (per $10^6$ cells): LA, 1.9; AA, 5.8; LNA, 2.7; EPA, 3.4; DHA, 3.1 μg (does not include exogenous FAs converted to other FAs of the same family). [c]Excluding sphingomyelin and lyso-PC, which were not analysed. [d]Any choline plasmalogens are included in this class since they are not separable by our method. Similarly, ethanolamine plasmalogens are included in the PE class. [e]Values in parentheses give the relative amount (mol%) of each phospholipid in MCF-7 cells as determined by phosphorus assay. Typically, sphingomyelin and lyso-PC constituted 5–6% and <1% of phospholipids respectively (data not shown). [f]Values in brackets give the relative amount of neutral lipids based on wt% of total (endogenous + exogenous) FA measured in each fraction by GC.

(Caroll & Hopkins, 1979; Hubbard & Erickson, 1987; Katz & Boylan, 1987; Pritchard *et al.*, 1989; Cave, 1991). While inhibition by AA appears to be paradoxical, it should be noted that corn oil contains very little AA. The differing effect of LA compared with AA and the less extensive inhibition by LNA compared with EPA and DHA suggest that differences also exist among FAs of the same family.

Wicha *et al.* (1979) evaluated the effect of FAs on the growth of primary normal rat mammary epithelial cells and rat mammary tumour cells induced by 7,12-dimethylbenz[α]anthracene. Both cell types were stimulated by LA in the concentration range 0.1–10 μg ml$^{-1}$ (0.36–36 μM). LNA also stimulated the growth of both cell types in the lower end of the concentrations studied (0.36–3.6 μM), although the

**Figure 4** Comparison of the effects of **a**, AA, **b**, LA, **c**, DHA and **d**, LNA presented as albumin-bound free FA (■) or in phospholipid liposomes (▨) on the growth of MCF-7 breast cancer cells. In both cases, the medium contained 0.5% FBS and 1.5 g l$^{-1}$ BSA. The cells were exposed to 30 μM total FA or 30 μM total PC and were counted after 7 days. Albumin-bound OA or liposomes containing 16:0 at the *sn*-1 and OA at the *sn*-2 position of PC were used as control and as a supplement in all cases with test FA or PC below 30 μM. Points, mean values relative to control for three separate experiments with albumin-bound FAs and two separate experiments with liposomes, each set up in triplicate wells: bars, s.e.m. Significantly different cell number compared with control: $^{*}P<0.05$, $^{**}P<0.01$.

**Table V** Comparison of the effects of two methods of supplementation on *n*-3 fatty acid incorporation into and processing by MCF-7 breast cancer cells at day 3 of exposure to various unsaturated fatty acids[a]

| | *Percentage of total fatty acid*[b] | | | | | |
|---|---|---|---|---|---|---|
| | *Albumin-bound* | | | *In liposomes*[c] | | |
| *Fatty acid* | *OA* | *LNA* | *DHA* | *OA* | *LNA*[d] | *DHA* |
| Saturated[c] | 20.1 | 25.1 | 35.0 | 30.5 | 34.3 | 29.8 |
| Monounsaturated | 71.3 | 25.5 | 29.7 | 61.3 | 37.0 | 54.9 |
| $18{:}3_{n-3}$ (LNA) | 0.1 | 28.7 | ND | ND | 14.1 | 0.1 |
| $18{:}4_{n-3}$ | ND[e] | ND | ND | ND | ND | ND |
| $20{:}3_{n-3}$ | ND | 8.2 | ND | ND | 4.3 | ND |
| $20{:}4_{n-3}$ | ND | 2.6 | 0.6 | ND | 2.2 | ND |
| $20{:}5_{n-3}$ (EPA) | ND | ND | 7.4 | ND | ND | 0.3 |
| $22{:}3_{n-3}$ | ND | 1.3 | ND | ND | 0.6 | ND |
| $22{:}4_{n-3}$ | ND | 2.1 | ND | ND | 2.1 | ND |
| $22{:}5_{n-3}$ | ND | ND | 2.1 | ND | ND | 0.2 |
| $22{:}6_{n-3}$ (DHA) | 0.1 | 0.1 | 20.0 | ND | ND | 7.2 |
| Total *n*-6 FAs | 2.3 | 2.2 | 2.4 | 1.6 | 1.4 | 2.0 |
| All unknown FAs[f] | 6.1 | 4.2 | 2.8 | 6.6 | 4.0 | 5.5 |

[a]Data reflect the results of one experiment for each EFA added at 30 μM EFA or PC; similar results were obtained in replicate experiments for each EFA. [b]Total fatty acid content in each treatment case was (i) for albumin-bound (per 10$^{6}$ cells): OA, 39.0; LNA, 41.9; DHA, 51.8 μg; and (ii) for liposomes (per 10$^{6}$ cells): OA, 56.5; LNA, 41.9; DHA, 54.5 μg. [c]Unless otherwise indicated the test FAs were supplied only at the *sn*-2 position of PC (with 16:0 at the *sn*-1 position). Liposomes also supplied 6 μM 1-palmitoyl-2-oleoyl-PS. [d]Dilinolenoyl PC. [e]ND, none detected (<0.1%). [f]This row represents the sum of the contents of all unassigned peaks which do not change appreciably under any treatment.

stimulation was substantially more pronounced in normal cells. In contrast, normal cells were inhibited by AA levels $>3\,\mu M$. While direct comparison of our results with those of Wicha *et al.* (1979) is not possible, in both studies mammary tumour cells were not inhibited by LA at concentrations for which other FAs were inhibitory.

Our results are in qualitative agreement with the effects of EPA, DHA and LA reported by Rose and Connolly (1989, 1990) for the human breast cancer cell lines MDA-MB-231 and MCF-7. DHA, and to a lesser extent EPA, inhibited MDA-MB-231 cell growth in a dose-dependent manner ($1.5-7.5\,\mu M$). However, LA was found to stimulate the growth of MDA-MB-231 and, to a lesser extent, MCF-7 cells. Stimulation of MDA-MB-231 cells was optimal at $0.75\,\mu g\,ml^{-1}$ ($2.7\,\mu M$), and was abolished at higher concentrations ($6-30\,\mu M$). Although Rose and Connolly (1990) noted a dose-dependent inhibition of MDA-MB-231 cell growth by OA concentrations above $3\,\mu M$, no such inhibitory effect was noted in our studies with MCF-7 (or MCF-10A) cells, even at $30\,\mu M$. The lack of an effect of OA on MCF-7 cell growth has also been reported by others, even for concentrations exceeding $100\,\mu M$ (Borrás & Leclercq, 1992). The different effect of OA in the two breast cancer cell lines may be due to differences in sensitivity to exogenous FA exposure. In our hands MDA-MB-231 cells are extremely sensitive to any FA addition above $10\,\mu M$, with AA, EPA and DHA all severely cytotoxic (unpublished observations).

The growth-inhibitory and sometimes cytotoxic effects of PUFAs on cancer cells are often explained in terms of the intracellular fatty acids' susceptibility to oxidation (Bégin *et al.*, 1986; Horrobin, 1989, 1990). It has recently been reported that breast cancer cells are more susceptible to PUFA peroxidation than are normal cells (Takeda *et al.*, 1992). We minimised oxidation of easily oxidisable PUFAs (with three or more double bonds) during preparation, storage and incubation in the extracellular milieu by supplying vitamin E ($100\,\mu M$; 3:1 molar ratio to FA) with the FA–BSA complexes. We measured the concentration of TBARSs in the culture medium as a crude indicator of the overall level of lipid peroxidation. In our system greater inhibition by the more highly unsaturated FAs cannot be attributed to end products of extracellular PUFA oxidation. The concentration of TBARSs in the culture medium never exceeded $0.5\,\mu M$, while growth-inhibitory effects of MDA have only been reported for levels above $100\,\mu M$ (Bird & Draper, 1980). Although we did not measure the extent of intracellular PUFA peroxidation, it is reasonable to expect that vitamin E (a potent inhibitor of lipid peroxidation; Cheeseman *et al.*, 1984) inhibited this reaction as well. In this regard it should be noted that, in the nude mouse model, antioxidants prevented the inhibitory effects of *n*-3 FAs on MCF-7 tumour cell growth (Gonzalez *et al.*, 1991). Therefore, it is likely that the inhibition of MCF-7 cell growth we observed with LNA, AA, EPA and DHA is due to factors other than lipid peroxidation.

Many animal cells are deficient in one desaturating enzyme, usually $\Delta^6$ or $\Delta^4$ (Dunbar & Bailey, 1975; Maeda *et al.*, 1978; Robert *et al.*, 1978). Extensive two-carbon elongations in the absence of appreciable $\Delta^4$, $\Delta^5$, $\Delta^6$ or $\Delta^7$ desaturations, as observed here for MCF-7 cells, have not been previously reported. Reduced rates or the complete absence of $\Delta^6$ desaturation has been described in rat tumours *ex vivo* (Bartoli *et al.*, 1980; Cheeseman *et al.*, 1984). Furthermore, many but not all transformed or malignant cell lines *in vitro* have a reduced capacity for $\Delta^6$ desaturation (Dunbar & Bailey, 1975; Maeda *et al.*, 1978; Iturralde *et al.*, 1990; Marra & de Alaniz, 1992; Naval *et al.*, 1993).

The relationship between $\Delta^6$ desaturation and cancer remains unclear, but Horrobin (1989, 1990) suggests that $\Delta^6$ desaturation plays a key role in human cancer and in breast cancer in particular. Lack of $\Delta^6$ desaturation may render cells unable to safely accommodate 6-desaturated EFAs. This may be responsible for the cancer cells' susceptibility to AA, EPA and DHA. In this regard it should be noted that $\gamma$-linolenic acid ($18{:}3_{n-6}$), also a 6-desaturated EFA, has been reported to exert cytotoxic effects on cancer cells from various tissues (Bégin *et al.*, 1986). In contrast to MCF-7 cells, MCF-10A cells are much more resistant to AA, EPA and DHA at concentrations below $30\,\mu M$, consistent with these cells' ability to produce substantial amounts of these FAs from LA and LNA. Although the distinct differences in EFA processing between the MCF-10A and MCF-7 cell lines may be related to tumour progression, further studies with a variety of normal and cancerous cell lines and primary cells are necessary to confirm this hypothesis. The possibility must be considered that MCF-10A cells desaturate exogenous FAs more extensively than MCF-7 cells because of their faster growth rate. At $30\,\mu M$ FA, however, MCF-10A cells were at least as inhibited as MCF-7 cells. We would expect that the main effect of doubling time on EFA metabolism would be observed between quiescent and actively dividing cells. The finding of Bandyopadhyay *et al.* (1987) that both growing and non-growing mouse mammary epithelial cells metabolise LA to AA and prostaglandin $E_2$ suggests that the differences in EFA processing between MCF-7 and MCF-10A cells are not the result of differences in growth rate. It must be emphasised that the differences in EFA processing may not account for the observed inhibition of MCF-7, but not MCF-10A, cell growth by the *n*-3 FAs and AA at concentrations below $30\,\mu M$.

The fact that AA, LNA and DHA supplied in PC liposomes were as inhibitory to MCF-7 cell growth as the albumin-bound free FAs even though only 25–50% as much EFA was incorporated suggests different mechanisms of growth inhibition for the two forms of EFA supplementation. Introduction of EFAs in PC liposomes may upset their distribution across the phospholipid classes. Indeed, the distribution of exogenous EFAs in phosphoglycerides obtained when the EFAs were presented in PC liposomes was different from that obtained with albumin-bound free FAs. For example, the ratio of PC-bound to PE-bound DHA increased from 0.4 to 1.0 when this FA was supplied as PC rather than bound to albumin. For AA, the same ratio increased from 1.0 to 1.6. Just how sensitive the cell is to such changes remains to be determined. However, despite the increased ratio of PC-bound to PE-bound exogenous EFA, addition of exogenous PC did not alter the relative amounts of total PC and PE in MCF-7 cells ($50.5\% \pm 1.2\%$ PC and $26.7\% \pm 2.6\%$ PE for the albumin method compared with $50.3\% \pm 1.2\%$ PC and $21.3\% \pm 2.1\%$ PE for the liposome method). In addition, examination of the FA profiles of individual phosphoglyceride classes of liposome-supplemented MCF-7 cells reveals that all are enriched in the test EFAs (data not shown). These results suggest that the cell attempts to regulate the total phospholipid distribution and the composition of each class.

Endothelial cells produce enzymes that oxidise lipoproteins on the cell surface (Steinberg *et al.*, 1989) and may be able to act on the PC of liposomes as well. Therefore, the possibility of extracellular effects of liposomes, such as enzyme-induced peroxidations of the unsaturated FAs at the cell surface, cannot be excluded. In this regard it should be noted that the final vitamin E content was $10\,\mu M$ in liposome experiments (1:3 molar ratio to PC), whereas in experiments with albumin-bound free FAs it was $100\,\mu M$ (3:1 molar ratio to FA). However, no increase in TBARS concentration was measured even under the most inhibitory liposome conditions. Furthermore, the observed inhibitions were not due to a cytotoxic effect, since cell viability never dropped below 99% and 2- to 3-fold expansion in cell number was obtained even with the most inhibitory liposomes.

This work was supported in part by NIH Grant R01CA49564-04 (TAV), NSF Grant BCS-9058416 (WMM) and contributions to W.M.M. from Eli Lilly and Co., Schering Plough Research and Abbott Laboratories. We are grateful to Dr Ming Liu and Wilfred Buchanan at the Department of Endocrinology of Rush Medical College for expert assistance with the lipid analyses and for many helpful comments, and to Mary Jo Harvey at the Department of Chemical Engineering of Northwestern University for assistance with cell culture and the growth experiments.

### References

ARMSTRONG, B. & DOLL, R. (1975). Environmental factors and cancer incidence and mortality in different countries, with special reference to dietary practices. *Int. J. Cancer.*, **15**, 617–631.

BANDYOPADHYAY, G.K., IMAGAWA, W., WALLACE, D. & NANDI, S. (1987). Linoleate metabolites enhance the *in vitro* proliferative response of mouse mammary epithelial cells to epidermal growth factor. *J. Biol. Chem.*, **262**, 2750–2756.

BARNES, D. & SATO, G. (1979). Growth of a human mammary tumour cell line in a serum-free medium. *Nature*, **281**, 388–389.

BARTOLI, G.M., BARTOLI, S., GALEOTTI, T. & BERTOLI, E. (1980). Superoxide dismutase content and microsomal lipid composition of tumours with different growth rates. *Biochim. Biophys. Acta*, **620**, 205–211.

BÉGIN, M.E., ELLS, G., DAS, U.N. & HORROBIN, D.F. (1986). Differential killing of human carcinoma cells supplemented with *n*-3 and *n*-6 polyunsaturated fatty acids. *J. Natl Cancer Inst.*, **77**, 1053–1062.

BIRD, R.P. & DRAPER, H.H. (1980). Effect of malonaldehyde and acetaldehyde on cultured mammalian cells: growth, morphology, and synthesis of macromolecules. *J. Toxicol. Env. Health*, **6**, 811–823.

BLIGH, E.G. & DYER, W.J. (1959). A rapid method of total lipid extraction and purification. *Can. J. Biochem. Physiol.*, **37**, 911–917.

BORRÁS, M. & LECLERCQ, G. (1992). Modulatory effect of nonesterified fatty acids on structure and binding characteristics of estrogen receptor from MCF-7 human breast cancer cells. *J. Receptor Res.*, **12**, 463–484.

BUEGE, J.A. & AUST, S.D. (1978). Microsomal lipid peroxidation. *Methods Enzymol.*, **52**, 302–310.

BUELL, P. (1973). Changing incidence of breast cancer in Japanese-American women. *J. Natl Cancer Inst.*, **51**, 1479–1483.

CAREAGA-HOUK, M. & SPRECHER, H. (1989). The effect of a fish oil diet on the fatty acid composition of individual phospholipids and eicosanoid production by rat platelets. *Lipids*, **24**, 477–481.

CARROLL, K.K. & HOPKINS, G.J. (1979). Dietary polyunsaturated fat versus saturated fat in relation to mammary carcinogenesis. *Lipids*, **14**, 155–158.

CAVE, W.T. Jr (1991). Dietary *n*-3 (ω-3) polyunsaturated fatty acid effects on animal tumorigenesis. *FASEB J.*, **5**, 2160–2166.

CHEESEMAN, K.H., BURTON, G.W., INGOLD, K.U. & SLATER, T.F. (1984). Lipid peroxidation and lipid antioxidants in normal and tumor cells. *Toxicol. Pathol.*, **12**, 235–239.

DUNBAR, L.M. & BAILEY, J.M. (1975). Enzyme deletions and essential fatty acid metabolism in cultured cells. *J. Biol. Chem.*, **250**, 1152–1153.

GONZALEZ, M.J., SCHEMMEL, R.A., GRAY, J.I., DUGAN, L. Jr, SHEFFIELD, L.G. & WELSCH, C.W. (1991). Effect of dietary fat on growth of MCF-7 and MDA-MB231 human breast carcinomas in athymic nude mice: relationship between carcinoma growth and lipid peroxidation product levels. *Carcinogenesis*, **12**, 1231–1235.

GUILBAUD, N.F., GAS, N., DUPONT, M.A. & VALETTE, A. (1990). Effects of differentiation-inducing agents on maturation of human MCF-7 breast cancer cells. *J. Cell Physiol.*, **145**, 162–172.

HORROBIN, D.F. (1989). Polyunsaturated fatty acids and human cancer. In *Carcinogenesis and Dietary Fat*, Abraham, S. (ed.) pp. 247–262. Kluwer Academic Publishers: Boston.

HORROBIN, D.F. (1990). Essential fatty acids, lipid peroxidation, and cancer. In *Omega-6 Fatty Acids: Pathophysiology and Roles in Clinical Medicine*, Horrobin, D.F. (ed.) pp. 351–377. Alan R. Liss: New York.

HUBBARD, N.E. & ERICKSON, K.L. (1987). Enhancement of metastasis from a transplantable mouse mammary tumor by dietary linoleic acid. *Cancer Res.*, **47**, 6171–6175.

IMAGAWA, W., BANDYOPADHYAY, G.K., WALLACE, D. & NANDI, S. (1989). Phospholipids containing polyunsaturated fatty acyl groups are mitogenic for normal mouse mammary epithelial cells in serum-free primary cell culture. *Proc. Natl Acad. Sci. USA*, **86**, 4122–4126.

ISCOVE, N.N. (1984). Culture of lymphocytes and hemopoitic cells in serum-free medium. In *Cell Culture Methods for Molecular and Cell Biology*, Barnes, D.W., Sirbasku, D.A. & Sato, G.H. (eds) pp. 169–185. Alan R. Liss: New York.

ITURRALDE, M., GONZALEZ, B. & PINEIRO, A. (1990). Linoleate and linolenate desaturation by rat hepatoma cells. *Biochem. Int.*, **20**, 37–43.

KAIZER, L., BOYD, N.F., KRIUKOV, V. & TRITCHLER, D. (1989). Fish consumption and breast cancer risk: an ecological study. *Nutrition & Cancer*, **12**, 61–68.

KAREY, K.P. & SIRBASKU, D.A. (1988). Differential responsiveness of human breast cancer cell lines MCF-7 and T47D to growth factors and 17β-estradiol. *Cancer Res.*, **48**, 4083–4092.

KATZ, E.B. & BOYLAN, E.S. (1987). Stimulatory effect of high polyunsaturated fat diet on lung metastasis from the 13762 mammary adenocarcinoma in female retired breeder rats. *J. Natl Cancer Inst.*, **79**, 351–358.

LIN, A.A., NGUYEN, T. & MILLER, W.M. (1991). A rapid method for counting cell nuclei using a particle sizer/counter. *Biotech. Techniques*, **5**, 153–156.

MAEDA, M., DOI, O. & AKAMATSU, Y. (1978). Metabolic conversion of polyunsaturated fatty acids in mammalian cultured cells. *Biochim. Biophys. Acta*, **530**, 153–164.

MARINETTI, G.V. (1962). Chromatographic separation, identification, and analysis of phosphatides. *J. Lipid Res.*, **3**, 1–20.

MARRA, C.A. & DE ALANIZ, M.J.T. (1992). Incorporation and metabolic conversion of saturated and unsaturated fatty acids in SK-$Hep_1$ human hepatoma cells in culture. *Mol. Cell Biochem.*, **117**, 107–118.

NAVAL, J., MARTINEZ-LORENZO, M.J., MARZO, I., DESPORTES, P. & PINEIRO, A. (1993). Alternative route for the biosynthesis of polyunsaturated fatty acids. *Biochem. J.*, **291**, 841–845.

PRITCHARD, G.A., JONES, D.L. & MANSEL, R.E. (1989). Lipids in breast carcinogenesis. *Br. J. Surg.*, **76**, 1069–1073.

ROBERT, J., REBEL, G. & MANDEL, P. (1978). Utilization of polyunsaturated fatty acid supplements by cultured neuroblastoma cells. *J. Neurochem.*, **30**, 543–548.

ROSE, D.P. & CONNOLLY, J.M. (1989). Stimulation of growth of human breast cancer cell lines in culture by linoleic acid. *Biochem. Biophys. Res. Commun.*, **164**, 277–283.

ROSE, D.P. & CONNOLLY, J.M. (1990). Effects of fatty acids and inhibitors of eicosanoid synthesis on the growth of a human breast cancer cell line in culture. *Cancer Res.*, **50**, 7139–7144.

ROSENTHAL, M.D. (1987). Fatty acid metabolism of isolated mammalian cells. *Prog. Lipid Res.*, **26**, 87–124.

ROSENTHAL, M.D., GARCIA, M.C., JONES, M.R. & SPRECHER, H. (1991). Retroconversion and $\Delta^4$ desaturation of docosatetraenoate (22:4(*n*-6)) and docosapentaenoate (22:5(*n*-3)) by human cells in culture. *Biochim. Biophys. Acta*, **1083**, 29–36.

SOULE, H.D., VAZQUEZ, J., LONG, A., ALBERT, S. & BRENNAN, M. (1973). A human cell line from a pleural effusion derived from a breast carcinoma. *J. Natl Cancer Inst.*, **51**, 1409–1416.

SOULE, H.D., MALONEY, T.M., WOLMAN, S.R., PETERSON, W.D. Jr, BRENZ, R., MCGRATH, C.M., RUSSO, J., PAULEY, R.J., JONES, R.F. & BROOKS, S.C. (1990). Isolation and characterization of a spontaneously immortalized human breast epithelial cell line, MCF-10. *Cancer Res.*, **50**, 6075–6086.

SPECTOR, A.A. & BURNS, C.P. (1987). Biological and therapeutic potential of membrane lipid modification in tumors. *Cancer Res.*, **47**, 4529–4537.

STEINBERG, D., PARTHASARATHY, S., CAREW, T.E., KHOO, J.C. & WITZTUM, J.L. (1989). Beyond cholesterol. Modifications of low-density lipoprotein that increase its atherogenicity. *N. Engl. J. Med.*, **320**, 915–924.

SUBBAIAH, P.V., KAUFMAN, D. & BAGDADE, J.D. (1993). Incorporation of dietary *n*-3 fatty acids into molecular species of phosphatidyl choline and cholesteryl ester in normal human plasma. *Am. J. Clin. Nutr.*, **58**, 360–368.

TAKEDA, S., HORROBIN, D.F., MANKU, M., SIM, P.G., ELLS, G. & SIMMONS, V. (1992). Lipid peroxidation in human breast cancer cells in response to gamma-linolenic acid and iron. *Anticancer Res.*, **12**, 329–333.

VOSS, A., REINHART, M., SANKARAPPA, S. & SPRECHER, H. (1991). The metabolism of 7, 10, 13, 16, 19-docosapentaenoic acid to 4, 7, 10, 13, 16, 19-docosahexaenoic acid in rat liver is independent of a 4-desaturase. *J. Biol. Chem.*, **266**, 19995–20000.

WICHA, M.S., LIOTTA, L.A. & KIDWELL, W.R. (1979). Effects of free fatty acids on the growth of normal and neoplastic rat mammary epithelial cells. *Cancer Res.*, **39**, 426–435.

WILLET, W.C., HUNTER, D.J., STAMPFER, M.J., COLDITZ, M.J., MANSON, J.E., SPIEGELMAN, D., ROSNER, B.A., HENNEKENS, C.H. & SPEIZER, F.E. (1992). Dietary fat and the risk of breast cancer. *JAMA*, **268**, 2037–2044.

WOODLE, M.C. & PAPAHADJOPOULOS, D. (1989). Liposome preparation and size characterization. *Methods Enzymol.*, **171**, 193–217.

WYNDER, E.L., ROSE, D.P. & COHEN, L.A. (1986). Diet and breast cancer in causation and therapy. *Cancer*, **58**, 1804–1813.

YEO, Y.K. & HOLUB, B.J. (1990). Influence of dietary fish oil on the relative synthesis of triacylglycerol and phospholipids in rat liver *in vivo*. *Lipids*, **25**, 811–814.